# Quality of life in patients with various Barrett's esophagus associated health states

**DOI:** 10.1186/1477-7525-4-45

**Published:** 2006-08-02

**Authors:** Chin Hur, Eve Wittenberg, Norman S Nishioka, G Scott Gazelle

**Affiliations:** 1 Gastrointestinal Unit, Massachusetts General Hospital, Boston, MA, USA; 2 Institute for Technology Assessment, Massachusetts General Hospital, Boston, MA, USA; 3 Department of Health Policy and Management (GSG), Harvard School of Public Health, Boston, MA, USA

## Abstract

**Background:**

The management of Barrett's esophagus (BE), particularly high grade dysplasia (HGD), is an area of much debate and controversy. Surgical esophagectomy, intensive endoscopic surveillance and mucosal ablative techniques, especially photodynamic therapy (PDT), have been proposed as possible management strategies. The purpose of this study was to determine the health related quality of life associated with Barrett's esophagus and many of the pivotal health states associated with Barrett's HGD management.

**Methods:**

20 patients with Barrett's esophagus were enrolled in a pilot survey study at a large urban hospital. The utility of Barrett's esophagus without dysplasia (current health state) as well as various health states associated with HGD management (hypothetical states as the subject did not have HGD) were measured using a validated health utility instrument (Paper Standard Gamble). These specific health states were chosen for the study because they are considered pivotal in Barrett's HGD decision making. Information regarding Barrett's HGD was presented to the subject in a standardized format that was designed to be easily comprehendible.

**Results:**

The average utility scores (0–1 with 0 = death and 1 = perfect health) for the various Barrett's esophagus associated states were: BE without dysplasia-0.95; Post-esophagectomy for HGD with dysphagia-0.92; Post-PDT for HGD with recurrence uncertainty-0.93; Post-PDT for HGD with recurrence uncertainty and dysphagia-0.91; Intensive endoscopic surveillance for HGD-0.90.

**Conclusion:**

We present the scores for utilities associated with Barrett's esophagus as well as various states associated with the management of HGD. The results of our study may be useful in advising patients and providers regarding expected outcomes of the various HGD management strategies as well as providing utility scores for future cost-effectiveness analyses.

## Background

Barrett's esophagus (BE) is a result of chronic reflux disease and is a risk factor for esophageal adenocarcinoma [1] following a proposed dysplasia-carcinoma sequence: intestinal metaplasia (BE); to low grade dysplasia (LGD); to high grade dysplasia (HGD); to adenocarcinoma. Daily symptoms of gastroesophageal reflux disease have been reported in 7% of the population [2] and is of public concern because of the alarming rise in esophageal adenocarcinoma incidence in the past two decades [3].

Although surgical esophagectomy is considered by many as the standard management for esophageal cancer in those patients who are operative candidates, a consensus regarding the optimal management of HGD does not exist. Publications have reported a wide range 27–73% [4-10] of missed and concomitant cancers when patients with HGD detected by endoscopic biopsy undergo surgical resection. Advocates of surgery have therefore proposed that all patients with HGD should undergo prophylactic esophagectomy [11]. However, the morbidity and mortality associated with surgical esophagectomy is of considerable concern [12]. Furthermore, the largest published study to date of more than 1000 patients with over a 7 year period of follow-up found that the 'missed' esophageal cancer rate in HGD was lower than previous reports [13], further arguing that the risks of surgery may outweigh the potential benefits and that endoscopic surveillance may be a reasonable strategy.

Mucosal ablation is an area of much current investigation and provides an intermediate option between surgery and endoscopic surveillance, with the most data available for photodynamic therapy (PDT). PDT is an endoscopic ablative treatment that has successfully treated patients with BE and early esophageal cancer or HGD who have traditionally been poor operative candidates for esophagectomy [14]. Although larger studies demonstrating PDT's long-term efficacy are not currently available, if proven effective, the low mortality and morbidity associated with PDT and the fact that patients can be treated on an outpatient basis make it an attractive potential first-line therapy of BE with HGD. Furthermore, a published cost-effectiveness analysis [15] suggested PDT could be a preferred strategy, but only if the quality of life after PDT was relatively high. The purpose of this pilot study was to determine the utility of health states associated with Barrett's esophagus and Barrett's HGD management, in order to elucidate the outcomes of different management strategies and inform clinical decision making. Utility assessment is a particularly appealing quality of life measure because it incorporates all aspect of health into a single number (between 0 and 1) with the extreme endpoints of death and perfect health [16].

## Methods

### Patients

Patients with documented (by histology) Barrett's esophagus over the age of 18 who were either having an endoscopy or a clinic visit within the Massachusetts General Hospital (large, urban hospital) GI Associates' practice were identified by one of the investigators using the practice's patient scheduling system. Subjects gave informed consent prior to participation and received no remuneration.

After permission was obtained from the patient's physician, the investigator invited the potential subject to participate. A total of 26 patients were asked to participate in this study and 20 completed the study. The institutional review board overseeing human research at the Massachusetts General Hospital approved the study.

Patients recruited in the endoscopy unit (18/20) were approached prior to their endoscopy, and if willing, a future telephone appointment was made to administer the questionnaire. The subject was also given written copies of the questionnaires (described in next section) in a packet to take home for review prior to the telephone call. Alternatively, if the subject was recruited in the outpatient clinic (2/20), the questionnaire was administered in person after the scheduled physician visit.

Regardless of the method used to administer the survey, the investigator attempted to standardize the interview as much as possible.

Patients with Barrett's esophagus were chosen for the study because they would be familiar with endoscopic surveillance and may have considered many of the issues regarding HGD management, thereby making them an informed and realistic patient population facing these decisions. Although patients with HGD or prior HGD were excluded, patients with prior LGD were included.

The description of the patient recruitment and separate data acquired from these recruited subjects have been previously published [17]. However, the data presented in this manuscript are the results of a new analysis using distinct data that have not yet been published (except in abstract form) [18].

### Study administration and materials

The standard gamble instrument is considered the gold standard method for utility measurement [19]. To assess utilities in our subjects with BE, we used a previously validated paper version of the standard gamble [16,20] (Appendix [see Additional file 1]).

After measuring the subjects' utility for their current health state (Barrett's esophagus without dysplasia), each enrolled patient was then asked to assess four hypothetical health states that might result from management strategies for Barrett's HGD. These states included: 1) Post-surgical esophagectomy for HGD without concern regarding BE or dysplasia recurrence but with dysphagia; 2) Post-successful PDT for HGD with concern about an unknown chance of recurrence but no dysphagia; 3) Post-successful PDT for HGD with concern about an unknown chance of recurrence and with dysphagia; 4) Intensive Endoscopic Surveillance (Appendix for complete descriptions of all the health states [see Additional file 1]). Each of these hypothetical health states was presented in a standardized format including risks of BE and HGD recurrence, future endoscopic surveillance regimens and possible morbidity or side effects.

Table 1 presents the estimates for various aspects of the strategies portrayed in the health state descriptions with references to the published literature upon which they are based. In constructing the descriptions of the various health states, a careful balance was sought between accurately portraying the medical complexities involved in each state and minimizing "cognitive burden" (i.e., effort required to perceive, think and remember) as described in Furlong et al.'s guide to health state questionnaires [21]. A summary in bullet format was provided for each health state to help the subject keep the important factors in mind while undergoing utility assessment.

**Table 1:** References for characteristics of health states

**Characteristic**	**Patient Simplified Description**	**Published Values**	**References**
**Esophagectomy**			
Sx Success Rate	"cured"	Recurrence 0–2%/year*	Rice [7], Ferguson [27]
Dysphagia Treatment	"3 endoscopies"		Headrick [28]
Endoscopic Dilation Perforation	"1 in 200"	>0.25%/dilation	Bueno [29]
*Follow-up Surveillance*			
EGD every year			Hur [30]
**Photodynamic Therapy**			
Recurrence Risk	"chance of recurrence"		Barham [31], Bonavina [32]
Dysphagia Treatment	"3 endoscopies"		Headrick [28]
Endoscopic Dilation Perforation	"1 in 200"	>0.25%/dilation	Bueno [29]
*Follow-up Surveillance*			
EGD every 3–6 months for 2 years and then yearly			Hur [15]
**Intensive Endoscopy**			
EGD every 3 months			Sampliner [33]

**Abbreviations**: EGD-upper endoscopy; Sx-surgery; PDT-photodynamic therapy.

In the standard gamble (SG) utility assessment method, patients are offered an option such as an imaginary pill that will result in either perfect health or death. The maximum amount of risk of death that a patient is willing to assume for a chance at perfect health is determined and used to derive the utility of the health state in question [22]. The SG instrument was originally administered face-to-face with trained interviewers, but the more recently developed Paper Standard Gamble was developed and validated so that instrument could be self-administered [16]. In our study, although the Paper Standard Gamble (Appendix [see Additional file 1]) was used, a study investigator provided each subject with directions regarding the instrument and allowed the patient to ask questions, either in person (2/20), or by telephone (18/20) during utility measurements for each health state presented. All surveys were administered by a single investigator (C.H.) who tried, if it all possible, to limit the number of questions asked by subjects during the interview, in an attempt to maintain study standardization.

On average, this portion of the questionnaire took approximately 15 minutes to complete. At the end of the interview, the interviewer qualitatively assessed the perceived quality of the subject's comprehension on a scale of 1–3 (1-poor, 2-fair, 3-excellent).

The subject's demographic and clinical data were retrieved from the patient's electronic medical record after the interview was completed. The study instruments and algorithm were tested and refined on four (non-patient) subjects for feasibility and comprehension prior to use with actual study subjects. The primary refinements that resulted from this 'pre-testing' were further simplifications of many of the medical terms used to describe the various health states.

### Data analysis

This was a descriptive, cross-sectional study where the results are presented as average (mean and median) scores with ranges and standard deviations. No statistical analyses or power calculations were performed for this pilot study.

## Results

### Clinical and demographic features

The mean age of the subjects in the study was 64.6 years and 55% (11/20) were male. 20% of the subjects had undergone a Nissen fundoplication surgery, 15% had a history of dysphagia and 10% had a history of Barrett's low grade dysplasia that subsequently regressed on follow-up endoscopic biopsies (Table 2).

**Table 2:** Patient Characteristics

**Characteristics**	**Mean**	**Range**
Age	64.6	49–77
Sex		
Male	55%	
Female	45%	
Prior Nissen		
Fundoplication	20%	
History of dysphagia	15%	
History of low grade dysplasia	10%	

### Questionnaire responses

The paper standard gamble utility scores are presented in Table 3. The average (mean) utility score for the subjects' actual health state (BE without dysplasia) was 0.95, with 0 representing death or the worst score and 1.0 representing the best score or perfect health. Utility scores elicited for various hypothetical health states related to the different management options associated with Barrett's HGD were as follows: Post-esophagectomy with dysphagia = 0.92; Post-PDT without dysphagia = 0.93; Post-PDT with dysphagia = 0.91. The state of undergoing intensive endoscopic surveillance as a management strategy for HGD resulted in a quality of life utility of 0.90. As would be expected, the utility scores for the HGD health states are lower (or worse quality of life) than the BE without dysplasia score.

**Table 3:** Utilities Associated with Various Barrett's Esophagus Health States

**Health State**	**Mean (Median)**	**Range**	**SD**
*Actual Patient Health State*			
Barrett's esophagus (no dysplasia)	0.95 (0.98)	0.775–0.995	0.067
*Hypothetical Patient Health States*			
Post-Esophagectomy with dysphagia	0.92 (0.96)	0.725–0.995	0.079
Post-PDT (no dysphagia)	0.93 (0.98)	0.550–0.995	0.107
			
Post-PDT with dysphagia	0.91 (0.96)	0.550–0.995	0.117
Intensive Endoscopic Surveillance	0.90 (0.95)	0.550–0.995	0.138

**Abbreviations**: PDT-photodynamic therapy.

The average rating of the interview quality or subject comprehension graded by the interviewer was 2.75 with all interviews rated either 2 or 3 (1–3 scale).

## Discussion

We present estimates of utilities for Barrett's esophagus as well as various health states associated with Barrett's HGD management and therapy using the Paper Standard Gamble instrument. Although other studies have analyzed quality of life in patients with gastroesophageal reflux disease (GERD) and BE, our analysis is the first to present utilities associated with many of the pivotal health states associated with Barrett's HGD management.

Gerson et al. recently published the results of an analysis which used a computer program to elicit utilities from patients with GERD as well as a subset of patients who also had BE [23]. The utilities derived from BE patients using the standard gamble were 0.95 for patients on reflux therapy and 0.93 for BE off of reflux therapy. Our BE without dysplasia utility score is within the same range as this separate and independent study, lending further credence to both analyses' findings. Another study of fifteen patients found that patients undergoing endoscopic surveillance reported a reduced quality of life distinct from their reflux symptoms [24]. Provenzale et al. [25] elicited utilities using the time-trade-off technique to estimate the quality of life after an esophagectomy and found a median value of 0.97 (or 97% of perfect health). However, no published analysis to date report utilities for the Barrett's HGD management states that we have studied

A limitation to the study was the relatively small sample size. This is of particular concern as large variations in quality of life were found among those who provided scores for BE. The congruency in our BE without dysplasia utility score and those of Gerson et al. [23] provides some reassurance, although the utilities elicited for the hypothetical states should be considered with some caution until confirmed in a larger study. We also chose to include patients who had a history of LGD, and although they only comprised 10% of the subjects studied, these patients could have a differing perspective of HGD.

Except for utilities scores for BE without dysplasia, the other utilities were evaluated for hypothetical states. Community or population utilities approximate societal values, which can be estimated by sampling general society. Especially if a disease or health state is rare, the societal value for a disease health state would be approximated by interviewing individuals who probably will not have the disease but would be asked to imagine a specific health state and then to assign a value to it using an instrument for this purpose [26]. In our study, the subjects were not a random sampling of general society, but of patients with BE without dysplasia. The hypothetical utilities derived from these subjects are somewhere between a population and patient perspective. We believe they were an appropriate group to study particularly because of their familiarity with Barrett's esophagus, endoscopy and esophageal adenocarcinoma.

Although our study subjects all had BE and some familiarity with many aspects of the hypothetical health states described, in order to present the information surrounding this clinical issue to participants who were presumed not to have prior medical training, it was necessary to simplify medical complexities to make it comprehendible and also to limit cognitive burden (see Methods section). The process of simplification could have theoretically led to biases, which could have influenced the participants' choices, which is a possible limitation to the study estimates for the hypothetical utility scores. The possibility of biases in these types of studies is, to a large part, unavoidable. However, the best efforts were made by the investigators to construct simplified presentations that were objective and based on published literature.

## Conclusion

Our study findings confirm the BE without dysplasia utility score previously reported [23] and provides utilities for pivotal health states associated in the management of Barrett's HGD. The results of this study can provide useful guidance for estimates to be used in cost-effectiveness analyses as well as guidance for designing larger Barrett's esophagus quality of life assessment studies. Our findings may also provide some preliminary data to aid both patient care providers and patients in the clinical decision making process regarding the optimal management of Barrett's HGD.

## Abbreviations

BE Barrett's esophagus;

EGD esophagogastroduodenoscopy;

HGD high grade dysplasia;

PDT photodynamic therapy.

## Competing interests

The author(s) declare that they have no competing interests.

## Authors' contributions

CH participated in the design, administration, statistical analysis, and manuscript preparation. EW participated in the design, statistical analysis and manuscript preparation. NSN and GSG contributed to study design and manuscript preparation.

## Supplementary Material

Additional File 1 Hur additional file. Appendix: 1. Paper Standard Gamble Survey; 2. Imagined Paper Standard Gamble; 3. Post Successful Esophagectomy with Dysphagia State Description; 4. Post Successful PDT (no dysphagia) Description; 5. Post Successful PDT with Dysphagia Description; 6. HGD Management with Intensive Endoscopic Surveillance Description Click here for file
