# Supplementary material for: Quality of life in patients with various Barrett's esophagus associated health states
Source: Health Qual Life Outcomes. 2006 Aug 2;4:45. doi: 10.1186/1477-7525-4-45 (PMC1559597; doi:10.1186/1477-7525-4-45)
Supplement: Additional File 1 — Hur additional file. Appendix: 1. Paper Standard Gamble Survey; 2. Imagined Paper Standard Gamble; 3. Post Successful Esophagectomy with Dysphagia State Description; 4. Post Successful PDT (no dysphagia) Description; 5. Post Successful PDT with Dysphagia Description; 6. HGD Management with Intensive Endoscopic Surveillance Description [file 1477-7525-4-45-S1.doc]

**Appendix,**

**Table of Contents**

1. Paper Standard Gamble Survey
2. Imagined Paper Standard Gamble
3. Post Successful Esophagectomy with Dysphagia State Description
4. Post Successful PDT (no dysphagia) Description
5. Post Successful PDT with Dysphagia Description
6. HGD Management with Intensive Endoscopic Surveillance Description

Paper Standard Gamble

Imagine a new (make-believe) pill is now available for ***all*** your health problems. Your doctor advises you that if you take the pill today and it works, it cures every health problem you ***currently*** have for the rest of your life. However, if you take the pill today and it ***does not*** work, it causes a sudden and painless death in your sleep tonight. Your doctor has no way of predicting which patients will be cured by this new (make-believe) pill, and will support whatever decision you make. Given everything you know about your current health, how it may change in the future, and your treatment options, we want to know what you think about this pill.

🡪***Would you take this pill right now if you knew***… (Please circle “Yes” or “No” for every question.)

…it had a **100%** chance of cure and a **0%** risk of causing death in your sleep tonight? **Yes No**

…it had a **99%** chance of cure and a **1%** risk of causing death in your sleep tonight? **Yes No**

…it had a **97%** chance of cure and a **3%** risk of causing death in your sleep tonight? **Yes No**

…it had a **95%** chance of cure and a **5%** risk of causing death in your sleep tonight? **Yes No**

…it had a **93%** chance of cure and a **7%** risk of causing death in your sleep tonight? **Yes No**

…it had a **91%** chance of cure and a **9%** risk of causing death in your sleep tonight? **Yes No**

…it had a **90%** chance of cure and a **10%** risk of causing death in your sleep tonight? **Yes No**

…it had a **85%** chance of cure and a **15%** risk of causing death in your sleep tonight? **Yes No**

…it had a **80%** chance of cure and a **20%** risk of causing death in your sleep tonight? **Yes No**

…it had a **75%** chance of cure and a **25%** risk of causing death in your sleep tonight? **Yes No**

…it had a **70%** chance of cure and a **30%** risk of causing death in your sleep tonight? **Yes No**

…it had a **65%** chance of cure and a **35%** risk of causing death in your sleep tonight? **Yes No**

…it had a **60%** chance of cure and a **40%** risk of causing death in your sleep tonight? **Yes No**

…it had a **50%** chance of cure and a **50%** risk of causing death in your sleep tonight? **Yes No**

…it had a **40%** chance of cure and a **60%** risk of causing death in your sleep tonight? **Yes No**

…it had a **30%** chance of cure and a **70%** risk of causing death in your sleep tonight? **Yes No**

…it had a **20%** chance of cure and a **80%** risk of causing death in your sleep tonight? **Yes No**

…it had a **10%** chance of cure and a **90%** risk of causing death in your sleep tonight? **Yes No**

…it had a **0%** chance of cure and a **100%** risk of causing death in your sleep tonight? **Yes No**

Paper Standard Gamble, Imagined Health Situation

Imagine a new (make-believe) pill is now available for ***all*** the health problems in this **imaginary situation**. Your doctor advises you that if you take the pill today and it works, it cures every health problem in this **imaginary situation** for the rest of your life. However, if you take the pill today and it ***does not*** work, it causes a sudden and painless death in your sleep tonight. Your doctor has no way of predicting which patients will be cured by this new (make-believe) pill, and will support whatever decision you make. Given everything you know about this **imaginary situation** and the treatments and risks involved, we want to know what you think about this pill.

🡪***Would you take this pill right now if you knew***… (Please circle “Yes” or “No” for every question.)

…it had a **100%** chance of cure and a **0%** risk of causing death in your sleep tonight? **Yes No**

…it had a **99%** chance of cure and a **1%** risk of causing death in your sleep tonight? **Yes No**

…it had a **97%** chance of cure and a **3%** risk of causing death in your sleep tonight? **Yes No**

…it had a **95%** chance of cure and a **5%** risk of causing death in your sleep tonight? **Yes No**

…it had a **93%** chance of cure and a **7%** risk of causing death in your sleep tonight? **Yes No**

…it had a **91%** chance of cure and a **9%** risk of causing death in your sleep tonight? **Yes No**

…it had a **90%** chance of cure and a **10%** risk of causing death in your sleep tonight? **Yes No**

…it had a **85%** chance of cure and a **15%** risk of causing death in your sleep tonight? **Yes No**

…it had a **80%** chance of cure and a **20%** risk of causing death in your sleep tonight? **Yes No**

…it had a **75%** chance of cure and a **25%** risk of causing death in your sleep tonight? **Yes No**

…it had a **70%** chance of cure and a **30%** risk of causing death in your sleep tonight? **Yes No**

…it had a **65%** chance of cure and a **35%** risk of causing death in your sleep tonight? **Yes No**

…it had a **60%** chance of cure and a **40%** risk of causing death in your sleep tonight? **Yes No**

…it had a **50%** chance of cure and a **50%** risk of causing death in your sleep tonight? **Yes No**

…it had a **40%** chance of cure and a **60%** risk of causing death in your sleep tonight? **Yes No**

…it had a **30%** chance of cure and a **70%** risk of causing death in your sleep tonight? **Yes No**

…it had a **20%** chance of cure and a **80%** risk of causing death in your sleep tonight? **Yes No**

…it had a **10%** chance of cure and a **90%** risk of causing death in your sleep tonight? **Yes No**

…it had a **0%** chance of cure and a **100%** risk of causing death in your sleep tonight? **Yes No**

# Post-Successful Esophagectomy with Dysphagia State

Imagine that you were found to have high grade dysplasia (or pre-cancerous tissue) in your esophagus and you had a procedure which cured you of the high grade dysplasia and your Barrett’s esophagus for the rest of your life. There is no chance of any recurrence.

You will now have endoscopy with biopsies every year just to check and make sure that you are fine.

As a result of the procedure you start experiencing a condition called dysphagia, which refers to the feeling of food sticking or getting hung up in the base of your throat or chest. It can often be painful, as well as annoying. It may be accompanied by pressure or pain in your chest. To treat this condition you will need to have on average 3 endoscopies performed where the area of narrowing in your esophagus that is causing these symptoms is dilated (expanded) so that you can swallow normally again. The dysphagia could return in the future requiring more endoscopic dilations. Also, for every 200 patients who have this dilation procedure, one gets a hole in his/her esophagus which would require emergency surgery to repair it.

You also have difficulty eating large meals because you feel full quickly and you lose weight as a result of this. You occasionally also notice heartburn after meals.

## Summary

- Cured of Barrett’s and dysplasia
- Endoscopy with biopsies every year
- Dysphagia treated by endoscopic dilations with risk of causing a hole
- Early fullness and weight loss, occasional heartburn

*Keep your imagined health situation in mind…*

# Post-Successful PDT without Dysplasia State

Imagine that you were found to have high grade dysplasia (or pre-cancerous tissue) in your esophagus and you had a procedure which successfully removed the high grade dysplasia and Barrett’s tissue. However, there is a chance of recurrence of the Barrett’s esophagus as well as the high grade dysplasia. There is also a chance that because of this treatment future biopsies of your esophagus taken during endoscopy may be less accurate, which could possibly delay a diagnosis of cancer, worsening the chance for a cure. The actual risk of recurrence or worsening the biopsy accuracy is not known. This uncertainty causes some people to worry a lot while others are fine with it.

After the procedure, you will have endoscopy with biopsies every 3-6 months for the first two years and then every year afterwards.

## Summary

- No more Barrett’s for now, but the chance for recurrence is unknown
- Biopsies may not be as accurate as before the procedure and a delay in cancer diagnosis could worsen chance for cure in the future
- Endoscopy with biopsy every 3-6 months for 2 years and then every year

*Keep your imagined health situation in mind…*

# Post-Successful PDT with Dysphagia State

Imagine that you were found to have high grade dysplasia (or pre-cancerous tissue) in your esophagus and you had a procedure which successfully removed the high grade dysplasia and Barrett’s tissue. However, there is a chance that the Barrett’s esophagus as well as the high grade dysplasia will recur. There is also a chance that because of this treatment the future biopsies of your esophagus taken during endoscopy may be less accurate, which could possibly delay a diagnosis of cancer, worsening the chance for a cure. The actual risk of recurrence or worsening the biopsy accuracy is not known. This uncertainty causes some people to worry a lot while others are fine with it.

After the procedure, you will have endoscopy with biopsies every 3-6 months for the first two years and then every year.

Also, as a result of the treatment you develop a condition called dysphagia, which refers to the feeling of food sticking or getting hung up in the base of your throat or chest. It can often be painful, as well as annoying. It may be accompanied by pressure or pain in your chest. To treat this condition you will need to have an average of 3 endoscopies performed where the area of narrowing in your esophagus that is causing these symptoms is dilated (expanded) so that you can swallow normally again. There is also a chance that the dysphagia could return in the future requiring more endoscopic dilations. Also, for every 200 patients who have this dilation procedure, one gets a hole in his/her esophagus which would require emergency surgery to repair it.

## Summary

- No more Barrett’s for now, but the chance for recurrence is unknown
- Biopsies may not be as accurate as before the procedure and a delay in cancer diagnosis could worsen chance for cure in the future
- Endoscopy with biopsy every 3-6 months for 2 years and then every year
- Dysphagia treated by endoscopic dilations with risk of causing a hole

*Keep your imagined health situation in mind…*

# Endoscopic Surveillance State

Imagine that you are found to have high grade dysplasia and that you will manage it by monitoring the condition with frequent endoscopy with biopsies to see if it turns into cancer.

You will have an endoscopy every 3 months with biopsies and have surgery if cancer is found in one of the biopsies.

You don’t know if your biopsies have missed a cancer or could miss one in the future possibly delaying surgery and worsening the chance for a cure. This uncertainty causes some people to worry a lot while others are fine with it.

## Summary

- No surgery or other invasive treatment for now
- Endoscopy with biopsy every 3 months
- Possible delay in cancer diagnosis could worsen chance for cure in the future

*Keep your imagined health situation in mind…*
